# Risk factors associated with experienced stigma among people diagnosed with mental ill-health: a cross-sectional study

**DOI:** 10.1007/s11126-020-09827-1

**Published:** 2020-08-28

**Authors:** C. Nugent, M. Rosato, L. Hughes, G. Leavey

**Affiliations:** Bamford Centre for Mental Health and Wellbeing; School of Psychology, Ulster University, Cromore Road, Coleraine, BT52 1SA Northern Ireland

**Keywords:** Mental illness, Stigma, Religiosity, Quality of Life, Trauma, Social Support

## Abstract

*Purpose:* to examine the relationship between religiosity, social support, trauma, quality of life and experienced stigma of mental illness amongst a population diagnosed with mental ill-health. *Methods:* A cross-sectional survey of day service users in Northern Ireland (*n* = 295) covering a range of issues including religiosity, social support, quality of life and prior experience of trauma. Stigma was measured using a recognised stigma scale. We used multinomial logistic regression to examine risk factors associated with experienced stigma. *Results:* Univariate analysis showed significant associations between stigma and age, number of friends, social support, quality of life and prior experience of trauma. Age, quality of life, and trauma remained independently associated with stigma in a multivariate logistic regression model (x^2^(12) = 98.40, *p* < 0.001). *Conclusion:* Younger people, those with less social support, prior experience of trauma and with poorer quality of life are at increased risk of experiencing stigma related to their diagnosis of mental illness. The findings provide further understanding of stigma and are useful for those overseeing programmes to improve access to mental health treatment.

## Introduction

Stigma and discrimination pose significant problems for people diagnosed with mental illness and may lead to *real-life* disadvantages, including poor access to mental and physical healthcare [1, 2], problems gaining or maintaining employment [3], difficulty accessing accommodation [4] and reduced life-expectancy [5, 6]. Mental illness may also impact negatively on stigmatised individuals by inducing *self-stigma*, self-judgement and reinforcement of negative stereotypes [7]. These can trigger low self-esteem [8], a *why-try* effect and loss of confidence when pursuing personal goals [5]. Self-perception of stigma amongst people with mental illness often leads to poorer attitudes towards psychotherapy [6], or even avoidance of professional help-seeking [9, 10] which in turn may lead to prolongation of symptoms and ultimately worse outcomes, including increased mortality.

## Factors associated with Stigma

Previous research suggests younger people report more mental illness-related stigma [11, 12], while those with higher levels of education are more likely to conceal their diagnosis [9]. Some studies show a gender effect but the evidence is inconsistent [10, 13–18]. Some evidence suggests a mediating role for social support, with increased peer support helping to challenge internalised stigma related to mental illness [19]. Conversely, a lack of support from public services or friends and family may be perceived as discrimination [20], while greater comfort in the ability to disclose mental illness to friends and family impacts positively on mental wellbeing [21]. Other studies have however found increased social support may be associated with a greater perception of stigma [22].

Similar ambiguity surrounds the impact of religiosity with some research [23] reporting the positive impact of religiosity on both physical and mental health, quality of life and life satisfaction, and another finding an association between religiosity and increased public stigma towards mental illness [24].

The relationship between trauma and stigma is well-established. Individuals exposed to traumatic events such as war or sexual abuse often feeling stigmatised because of their experiences, leading to feelings of shame and negatively impacting their ability to seek professional help. A recent study amongst refugee men in Australia for example found PTSD severity was associated (indirectly via mental health stigma) with lower help-seeking intentions [25]. Similarly, Amone-P’Olak et al [26], in their analysis of survivors of sexual violence during war in Northern Uganda found those reporting sexual abuse perceived more stigma and reported more barriers to seeking care than their peers without such experiences. Conversely, Andresen & Blais [27] found higher self-stigma was associated with a lower likelihood of disclosing military sexual trauma among female veterans.

People who attend day centres may be at increased risk of experiencing mental illness-related stigma. Their attendance at the centre makes it more difficult to conceal their diagnosis and may be itself associated with stigma independent of a diagnosis of mental illness. This population have been traditionally neglected in stigma research and little is known about the factors that may exacerbate or protect them against the negative effects of the stigma that surrounds their diagnosis of mental illness.

This study examines the relationship between religiosity, social support, trauma, quality of life and experienced stigma of mental illness amongst a population diagnosed with mental ill-health who attend a day centre in Northern Ireland.

## Method

### Sample

Stratified sampling was used to select participants with mental health problems (*n* = 504) within day centres across Northern Ireland (NI) (*n* = 14). Of those selected, 61% consented to participate (*n* = 306) and were provided with information sheets. Ethical approval was granted by the Office for Research Ethics Committees Northern Ireland (ORECNI). All data was anonymised prior to analysis.

### Data Collection

A comprehensive questionnaire was designed for use in the study and included questions related to socio-demographic characteristics and religiosity. The questionnaire also included established data-collection instruments - the Stigma Scale [28]; the Lancashire Quality of Life Profile (LQoLP) [29]; the Medical Outcomes Study (MOS) Social Support Survey [30]; and the Brief Trauma Questionnaire (BTQ) [31].

### Instruments

***Stigma*** was measured using the Stigma Scale [28], a twenty-eight item five-point Likert-type questionnaire designed to measure stigma associated with mental illness as perceived by service users, it includes components on positive and negative aspects of stigma, and internalization. Scores (global stigma) range from 0 to 112 with higher scores indicating more feelings of stigma and includes three subscales- *discrimination*, *disclosure* and *putative positive aspects* of mental illness. The scale was originally tested in a North London population of people diagnosed with mental illness and was shown to have both good internal reliability (Chronbach’s α = 0.87) and test-retest reliability (κ coefficients ranged from 0.4–0.71).

#### Quality of Life

The LQoLP [29] is a 105-item measure of Quality of Life using a seven-point scale (where higher scores indicate better QoL/satisfaction). It measures global well-being along nine life domains - work, leisure, religion, finance, living situation, legal and safety issues, family relations, social relations and health. An overall Quality of Life score was generated by taking an average of scores on each of the subscales.

***Social Support*** was measured using the MOS Social Support Survey [30], a 20-item measure covering four dimensions - emotional/informational, tangible, affection and positive social interaction – and a summary overall functional social support index. For this study only the overall index was used - with scores ranging from one to five (from lower to higher social support respectively).

***Trauma*** was measured using eight items from the Brief Trauma Questionnaire (BTQ) [31], a ten-item questionnaire measuring exposure to traumatic experiences, including violence towards the participant or a family member, unwanted sexual contact, involvement in a serious accident, and suffering a life-threatening illness. Items measuring exposure to war or war-related injuries and major natural or technological disasters were excluded. Scores range from zero to eight with higher scores reflecting greater exposure to traumatic events.

### Data Analysis

All analysis was carried out in Stata 13 [32]. Participants aged over seventy-five years (*n* = 6) and those for whom age was unknown (*n* = 5) were excluded from analysis. Missing values in the responses to the twenty-eight items of the Stigma Scale were imputed using the Stata Mi command [35, [33]. A summary stigma score (0–122) was generated by summing these items, and further summarised into four evenly distributed categories (0–46, 47–56, 57–66 and 67–112) - v*ery low*, *low*, *medium* and *high* respectively.

Descriptive statistics summarised the demographic characteristics of the sample, and logistic regression examined their association with stigma. Significantly associated variables (p < =0.05) were then included in a final multinomial logistic regression analysis (using the Stata mlogit macro): in these analyses independent variables no longer retaining significance in the fully adjusted model were dropped, and a subsequent model estimated.

## Results

After exclusions 295 people participated. Table 1 shows the age and gender breakdown by levels of stigma. Mean age was 51.8 years (SD = 10.2, range = 23–73) and 52% were female (*n* = 153). Global stigma scores ranged from 20 to 95 (mean = 56.6, SD = 13.7) with females scoring higher than males (mean = 57.2, SD = 13.8 and 55.7, SD = 13.6 respectively) and those in the youngest age-group scoring highest (mean = 63.7, SD = 16.32). Stigma scores generally decreased with increasing age: for males 53% and 5% reported high stigma levels in the youngest and oldest age groups respectively; and similarly, for females 47% and 12% reported high levels of stigma. Table 2 shows 16% (*n* = 47) of participants were *very religious*, 53% (*n* = 155) reported being *single or unmarried* and 51% (*n* = 129) reported having *five or more close friends and/or relatives.* Scores on the social support index ranged from 1 to 5 with a mean of 3.36 (SD = 0.92), overall Quality of Life scores ranged from 1.98–7.00 (mean = 5.16, SD = 0.77) and scores on the BTQ ranged from 0 to 8 with a mean of 1.96 (SD = 1.79).

**Table 1:** Global Stigma Scale scores by stigma category, gender and age group

Stigma Score	Males (*n* = 142)				Females (n = 153)			
	23–39	40–49	50–59	60+	23–39	40–49	50–59	60+
	*%* (n)				*%* (n)			
Very Low (0–46)	21 (4)	23 (10)	26 (11)	35 (13)	13 (2)	19 (8)	16 (9)	39 (16)
Low (47–56)	5 (1)	16 (7)	37 (16)	41 (15)	20 (3)	17 (7)	25 (14)	27 (11)
Medium (57–66)	21 (4)	33 (14)	19 (8)	19 (7)	20 (3)	36 (15)	27 (15)	22 (9)
High (67–112)	53 (10)	28 (12)	19 (8)	5 (2)	47 (7)	29 (12)	31 (17)	12 (5)
Total	19	43	43	37	15	42	55	41

**Table 2:** Sociodemographic and other characteristics of the sample

Characteristics	Category	*%* (n)
Age	23–39	11.5 (34)
	40–49	28.8 (85)
	50–59	33.2 (98)
	60+	26.4 (78)
Gender	Female	51.9 (153)
Religiosity	Very Religious	16.3 (47)
Marital Status	Married/Co-habiting	20.5 (60)
	Single/Unmarried	52.9 (155)
	Divorced/Separated	21.8 (64)
	Widowed	4.5 (14)
Number of Friends	Five or more	50.8 (129)
MOS Social Support Index	Mean (SD)	3.36 (0.92)
Quality of Life	Mean (SD)	5.16 (0.77)
BTQ Trauma	Mean (SD)	1.96 (1.79)

### Health and socio-psychological factors related to stigma

Table 3 shows results of a univariate logistic regression analysis comparing those recording the higher levels of stigma with those reporting very low levels. Generally, older people were less likely to report low (RR = 1.00, 95% CI = 0.97, 1.04), medium (RR = 0.96, 0.92, 0.99) and high (RR = 0.93, 0.89, 0.96) stigma levels than those younger. Females were associated with increased risks of reporting medium (57–66) (RR = 1.38, 0.72, 2.64) or high stigma (67–112) (RR = 1.39, 0.73, 2.67), though the effects were not statistically significant. Participants who were very religious (*n* = 47) were less likely to report a low stigma score (RR = 0.46, 0.15, 1.43), and increased likelihood of reporting a high score (RR = 1.99, 0.84, 4.71). However, neither were statistically significant. With marital status - when compared to those married/co-habiting, all other groups recorded higher likelihoods for reporting stigma: those widowed had a 31% excess risk (0.31, 5.62) of reporting a medium stigma score while all other groups displayed decreased risk. Conversely, those widowed displayed a decrease in risk of reporting a high stigma score while all other groups showed an increase. Those single/unmarried showed the greatest increases in risk (RR = 1.66, 0.66, 4.16) but again no result was statistically significant. Having five or more close friends or relatives had a protective effect across all stigma levels - risk of reporting a medium stigma score was reduced by ~60% (RR = 0.39, 0.19, 0.81) and risk of reporting a high score by 76% (RR = 0.24, 0.11, 0.50) compared to those with less than five close friends/relatives.

**Table 3:** Univariate analysis of stigma by category and independent variables

Independent Variable		Stigma^		
		(Very low vs) Low	(V. low vs) Medium	(V. low vs) High
		RR (95% CI)	RR (95% CI)	RR (95% CI)
Age	Continuous	1.00 (0.97, 1.04)	0.96 (0.92, 0.99)**	0.93 (0.89, 0.96)***
Gender	Females	0.97 (0.51, 1.86)	1.38 (0.72, 2.64)	1.39 (0.73, 2.67)
Religiosity	Very	0.46 (0.15, 1.43)	1.57 (0.66, 3.78)	1.99 (0.84, 4.71)
Marital Status	Married/Co-habiting	1.00 (ref)	1.00 (ref)	1.00 (ref)
	Single/Unmarried	0.64 (0.28, 1.47)	0.85 (0.36, 1.99)	1.66 (0.66, 4.16)
	Divorced/Separated	0.74 (0.28, 1.95)	0.88 (0.32, 2.37)	1.31 (0.45, 3.84)
	Widowed	0.35 (0.06, 2.18)	1.31 (0.31, 5.62)	0.70 (0.11, 4.59)
Number of Friends	Five or more	0.48 (0.23, 1.00)	0.40 (0.19, 0.81)*	0.24 (0.11, 0.50)***
MOS Social Support	Continuous	0.80 (0.55, 1.16)	0.56 (0.39, 0.82)**	0.55 (0.38, 0.79)**
Quality of Life	Continuous	0.45 (0.26, 0.79)**	0.27 (0.16, 0.48)***	0.14 (0.08, 0.25)***
BTQ Trauma	Continuous	1.02 (0.81, 1.27)	1.46 (1.18, 1.80)***	1.69 (1.36, 2.01)***

^Very Low (0–46) category is reference group

*P = <0.05, **P = <0.01, ***P = <0.001

The Social Support Index showed a clear relationship with stigma: when compared to *very low* stigma levels, higher levels showed an inverse relationship with social support - RR = 0.80: 95%CI = 0.55, 1.16; RR = 0.56, 0.39, 0.82; and RR = 0.55: 0.38, 0.79 for low, medium and high levels respectively. *Quality of Life* scores also significantly reduced the association with stigma. Each unit increase in *Quality of Life* was associated with a reduced risk of experiencing stigma. When compared to *very low* stigma levels - RR = 0.45: 95%CI = 0.26, 0.79 (low); RR = 0.27: 0.16, 0.48 (medium) and RR = 0.14: 0.08, 0.25 (high stigma). Finally, those with a greater history of trauma (BTQ) experienced more stigma: compared with *very low* stigma levels, a greater history of trauma were associated with increases of 46% (RR = 1.46, 1.18, 1.80) and 69% (RR = 1.69, 1.36, 2.01) in *medium* and *high* levels of stigma respectively.

### Multivariate Analysis

Indicators associated with stigma at univariate level (p < =0.05) were included in the multivariate analysis (Table 4). In each case Model 1 (M1) shows the fully adjusted model. Quality of life and trauma remained statistically significant predictors of stigma after adjusting for age, gender, number of friends, social support and each other. Older participants were associated with a small risk reduction (5%) for reporting high levels of stigma (RR = 0.95, 95%CI = 0.91, 0.99) while those reporting good quality of life showed a risk reduction of around 84% (RR = 0.16, 0.08, 0.35). Increased exposure to traumatic events was associated with an excess risk of 39% for reporting high levels of stigma (RR = 1.39, 1.07, 1.81).

**Table 4:** Multinomial logistic regression models of associated independent variables on relative risk of stigma per category compared to very low stigma (0–46)

Included Variables	Low (47–56)		Medium (57–66)		High (67–112)	
	M1	M2	M1	M2	M1	M2
	RRR (95% CI)	RRR (95% CI)	RRR (95% CI)	RRR (95% CI)	RRR (95% CI)	RRR (95% CI)
Age	1.01 (0.97, 1.05)	1.01 (0.97, 1.04)	0.98 (0.94, 1.02)	0.97 (0.93, 1.00)	0.95 (0.91, 0.99)^*^	0.94 (0.90, 0.98)^**^
Gender (female)	0.64 (0.31, 1.33)	0.88 (0.45, 1.73)	1.15 (0.53, 2.49)	1.28 (0.64, 2.58)	1.26 (0.55, 2.88)	1.29 (0.60, 2.76)
Number of Friends (5+)	0.60 (0.27, 1.31)		0.76 (0.33, 1.75)		0.70 (0.29, 1.73)	
Quality of Life	0.42 (0.21, 0.85)^*^	0.42 (0.24, 0.75)^**^	0.31 (0.15, 0.64)^**^	0.34 (0.19, 0.61)^***^	0.16 (0.08, 0.35)^***^	0.18 (0.10, 0.34)^***^
MOS Social Support Index	1.05 (0.67, 1.66)		0.82 (0.51, 1.33)		0.96 (0.57, 1.62)	
BTQ Trauma	1.02 (0.78, 1.32)	0.96 (0.76, 1.21)	1.38 (1.07, 1.77)^*^	1.30 (1.04, 1.62)^*^	1.39 (1.07, 1.81)^*^	1.37 (1.08, 1.73)^**^

*P = <0.05, **P = <0.01, ***P = <0.001

To estimate a second model (M2) - variables non-significant in M1 were removed leaving age, gender, quality of life and trauma. Increasing age was associated with a further risk reduction compared with M1 (RR = 0.94, 95%CI = 0.90, 0.98) while females showed excess risk of reporting the highest level of stigma compared to males (RR = 1.29, 0.60, 2.76), though this was not statistically significant. Those reporting better quality of life remained at less risk (RR = 0.18, 0.10, 0.34) but the effect was slightly smaller than in the previous model, and similarly those reporting greater exposure to traumatic events had a greater risk of reporting high levels of stigma but less so than in M1 (RR = 1.37, 1.08, 1.73). Comparison of the models showed little difference overall. Relative risks for individual variables change only marginally between M1 and M2, with the largest changes noted for; gender and low (RR = 0.64 to RR = 0.88), and medium (RR = 1.15 to RR = 1.28) stigma scores.

## Discussion

People with mental illness require appropriate support to help manage their condition. Stigma associated with mental illness presents significant barriers to this and presents practical, real-life problems for people with mental illness - for example, in finding and keeping work [3], accessing accommodation [4] and preventing those with mental illness from seeking professional help [34]. This study examines the relationship between stigma associated with mental illness and selected demographic and psychosocial factors. The Stigma Scale [28] measured stigma as perceived by people diagnosed with a mental illness, providing insight into how stigma is experienced by those directly affected. Participants were mostly aged over forty years, unemployed and either single or unmarried. Those at highest risk of feeling stigmatised were younger, had fewer than five close friends/relatives, poorer social support, had suffered greater exposure to traumatic events and generally reported poorer quality of life.

While the literature around stigma and age is ambiguous the findings reported here – that younger people were more likely to report stigma than their older counterparts - are consistent with other studies [11, 17, 28]. Oshodi et al [12] suggests this may be explained by the increased importance placed on dating and intimate relationships by younger people who are more likely to be in the process of starting their own families. They also suggest that younger respondents are perhaps more likely to have experienced unfair treatment at work because of their illness, when compared with older respondents who may have already attained senior positions in their workplace. Patten et al [35] suggests that the experience of stigmatisation may diminish over time as people learn to cope.

Similar to other studies [9, 12, 35, 36], females displayed greater risk (~40%) of experiencing stigma (both medium and high levels) than males, although this was not statistically significant.

Increased social support and having more than five close friends or relatives were associated with decreased risk of experiencing stigma in the univariate analysis. This is consistent with a number of other studies including Rusch et al [21], Robinson et al [37] and Moore & Ayers [22]- who found that use of internet forums by women with post-natal mental illness enabled them to disclose their illness to others. This raises an interesting question for future work in terms of the distinction between social support from peers with a diagnosis of mental illness themselves, and those without mental illness. In spite of this however, neither association held up when entered into the multivariate regression models.

Previous experience of trauma was strongly associated with stigma and the finding remained statistically significant after adjusting for age, gender, social support and quality of life. This is consistent with previous studies [38, 39], [40], although it is unclear from the current findings whether the stigma experienced by those with a previous exposure to trauma is related solely to their subsequent diagnosis of mental illness or to the traumatic event itself. A further analysis of stigma experienced as a direct result of trauma may help better understand this association.

A strong-negative association was found between quality of life and experience of stigma, similar to other studies; [44, [41–44]. It is not possible from this analysis to infer the direction of the observed association and it seems intuitive that either could precede the other. For example, it seems intuitive that being surrounded by less stigma (and better social support) helps ensure a better quality of life for people with a diagnosis of mental illness. Alternatively, it is possible that individuals who report less stigma may have a better quality of life to begin with and a more positive outlook as a result, mediating the amount of stigma that surrounds them. A similar question was posed by Holubova et al. as a result of their analysis of self-stigma and quality of life [45]. ﻿Perhaps key to the relationship between stigma and quality of life is the process of self-stigma, the internalising of stigmatised beliefs towards oneself. Both Hsiao et al [42] and Huang et al [46] highlight the mediating role of self-stigma through it’s impact on family coherence and individual quality of life, although both also emphasise the context of culture.

### Limitations

The study sample comprised a specific group of people diagnosed with mental illness who attended a day centre in Northern Ireland. Participants were mostly aged over forty years, unemployed and single or unmarried, making it difficult to measure differences across the full age spectrum and estimate the effects of socioeconomic circumstances and health inequalities. Stigma was as experienced by people diagnosed with mental illness and is not therefore a measure of stigmatising beliefs held by the general population. It may also be likely that, since study participants have actively sought help, they may represent those who experience less stigma or are less acutely impacted by it than people who avoid seeking help altogether. Thus, levels of reported stigma may underestimate that experienced by the wider population of people living with mental illness. Finally, the cross-sectional design makes it impossible to infer causation in any of the associations reported in this study.

## Conclusions

People with mental illness are often stigmatised because of their diagnosis which can, in turn, lead to a number of real-world disadvantages and negative outcomes. Those who are younger, with fewer friends and less social support, and who have been exposed to past trauma, experience greater risk of being stigmatised, making it potentially more difficult for such individuals to access adequate support. They also experience poorer quality of life. Interventions and programmes aimed at improving access to mental health treatment should take into account the additional complexity of stigma among such groups. The study raises questions for future work- including a comparison of the experience of stigma across mental health diagnoses, and further examination of the relationship between stigma related to mental illness and stigma related to trauma - such as sexual abuse - for individuals who have experienced both.
